# Responses of Soil Microbial Metabolic Activity and Community Structure to Different Degraded and Restored Grassland Gradients of the Tibetan Plateau

**DOI:** 10.3389/fpls.2022.770315

**Published:** 2022-04-08

**Authors:** Dangjun Wang, Huakun Zhou, Juan Zuo, Peng Chen, Yandi She, Buqing Yao, Shikui Dong, Jianshuang Wu, Fan Li, Denis Mburu Njoroge, Guoxi Shi, Xufeng Mao, Li Ma, Zhonghua Zhang, Zhun Mao

**Affiliations:** ^1^Key Laboratory of Aquatic Botany and Watershed Ecology, Wuhan Botanical Garden, Chinese Academy of Sciences, Wuhan, China; ^2^Key Laboratory of Cold Regions Restoration Ecology, Qinghai Province, Northwest Institute of Plateau Biology, Chinese Academy of Sciences, Xining, China; ^3^Sino-Africa Joint Research Center, Chinese Academy of Sciences, Wuhan, China; ^4^University of Chinese Academy of Sciences, Beijing, China; ^5^School of Grassland Science, Beijing Forestry University, Beijing, China; ^6^Institute of Environment and Sustainable Development in Agriculture, Chinese Academy of Agricultural Sciences, Beijing, China; ^7^Key Laboratory of Utilization of Agriculture Solid Waste Resources, College of Bioengineering and Biotechnology, Tianshui Normal University, Tianshui, China; ^8^School of Geographical Sciences, Qinghai Normal University, Xining, China; ^9^AMAP, Univ. Montpellier, CIRAD, CNRS, INRAE, IRD, Montpellier, France

**Keywords:** alpine grassland, microbial community, soil properties, degradation gradient, artificial grassland establishment

## Abstract

Climate change and land-use disturbances are supposed to have severely affected the degraded alpine grasslands on the Tibetan Plateau. Artificial grassland establishment has been implemented as a restoration tool against grassland degradation. However, the impact of such degradation and restoration processes on soil microbial communities and soil quality is not clearly understood. Here, we aim to investigate how the dynamics of microbial community and soil quality of alpine grasslands respond to a gradient of degradation and that of restoration, respectively. We conducted a randomised experiment with four degradation stages (light, moderate, heavy, and extreme degradation) and three restoration stages (artificial restoration for 1, 5, and 10 years). We analysed the abundance and diversity of soil bacteria and fungi, and measured soil nutrients, enzymatic activity and microbial biomass. The concentration of soil nitrogen (TN), soil organic matter (OM) in heavy degraded grassland decreased significantly by 37.4 and 45.08% compared with that in light degraded grassland. TN and OM in 10-years restored grassland also increased significantly by 33.10 and 30.42% compared to that in 1-year restored grassland. Four soil enzymatic activity indicators related to microbial biomass decreased with degradation gradient and increased with recovery time (i.e., restoration gradient). Both bacterial and fungal community structure was significantly different among grassland degradation or restoration successional stages. The LEfSe analysis revealed that 29 fungal clades and 9 bacterial clades were susceptible to degraded succession, while16 fungal clades and 5 bacterial clades were susceptible to restoration succession. We conclude that soil quality (TN, OM, and enzymatic activity) deteriorated significantly in heavy degraded alpine grassland. Soil microbial community structure of alpine is profoundly impacted by both degradation and restoration processes, fungal communities are more sensitive to grassland succession than bacterial communities. Artificial grasslands can be used as an effective method of restoring degraded grassland, but the soil functions of artificial grassland, even after 10 years of recovery, cannot be restored to the original state of alpine grassland.

## Highlights

- Soil nitrogen and carbon decreased significantly in heavy degraded alpine grassland.
- Soil enzymes related to microbial biomass changed significantly in grassland succession.
- Fungal communities were more sensitive to grassland succession than bacterial communities.
- After 10 years, soil function was not yet restored to pre-degradation levels.

## Introduction

Alpine grasslands take a tremendous area for livestock production in China and are served as an important barrier for ecological security of water conservation and carbon sequestration (Dong et al., 2020; Wang et al., 2020). Such a world unique ecosystem is extremely sensitive to global climate change (Li et al., 2019). There are approximately 5,000 km^2^ of degraded alpine grasslands on the Tibetan Plateau, accounting for a third of the total grassland area (Cui and Graf, 2009; Zhang et al., 2013). Moreover, the long-term overgrazing and unsustainable use have rapidly deteriorated alpine grasslands in terms of structure and function (Saruul et al., 2019; Wang et al., 2020), including soil nutrient loss, intensified soil erosion, decreased vegetation distribution and productivity, and loss of ecosystem services (Saruul et al., 2019). The degradation of alpine grassland is a progressive process spanning from light degradation, moderate degradation, and severe degradation, to extreme degradation or “black-soil land” according to assessment of plant coverage (Liu et al., 2017; Supplementary Table 1). The most seriously degraded grasslands refer to the phenomenon of “black soil land,” which accounts for about 16.5% of the degraded grassland area. These “black soil lands”, are characterised by low vegetation coverage (<30%) and biodiversity, declined herbage yield and loss of the upper soil horizons (Wang et al., 2006). Therefore, it is important to restore these grassland landscapes to their natural and original state. Conventionally used actions include livestock control, grazing exclusion, fencing pasturelands, and nutrients addition (Akiyama and Kawamura, 2007). However, none of these practices have proved to show positive effects on restoring the extremely degraded “black soil land” grasslands of the Tibetan Plateau (Wu et al., 2010; Li et al., 2014). Under such a context, artificial grasslands with *Elymus nutans* and *Poa pratensis* have been established in Guoluo Prefecture of Qinghai Province as an alternative approach (Li et al., 2014; Gao et al., 2021). *E. nutans* is the dominant grass species of these artificial grasslands (Dong et al., 2015).

Preliminary studies have considered artificial grassland establishment a promising, even an indispensable practice against severe grassland degradation, especially “black soil lands.” (Li et al., 2014). Wang et al. (2005) reported that artificial grassland establishment could improve the concentration of carbon and nitrogen in alpine meadows of the Tibetan plateau. Wu et al. (2010) found that concentrations of soil organic matter, nitrogen, and phosphorus in the “black soil lands” at the eastern Tibetan plateau increased significantly in the three- and six-year artificial grasslands. Nevertheless, Li et al. (2014) demonstrated that the concentration of soil nutrients substantially decreased with the increase in planting years of the alpine grasslands. Dong et al. (2015) also found that soil carbon in the five- year artificial grasslands was higher than in seven- and nine- year artificial grasslands in the extremely degraded case. Consequently, no consensus has been achieved regarding the changes in soil carbon content in artificial grasslands at different stages of succession and the underlying mechanism is not yet clear.

Soil microbial features, such as biodiversity, community structure and enzyme activity, are important biological characteristics of soil quality and deeply impact grassland ecosystem services (Dong et al., 2015; Karimi et al., 2017). Soil microorganisms (e.g., bacteria and fungi) are key drivers in the transformation of plant-soil nutrients, organic carbon and nitrogen cycling, pollutant degradation, and other soil biochemical reaction processes (Korshunova et al., 2019; Jansson and Hofmockel, 2020). Their high biochemical activities make them a reservoir and source of soil available nutrients (Jansson and Hofmockel, 2020). Soil microorganisms play an essential role in grassland succession (Cai et al., 2014; Luo et al., 2020). So far, no study has systematically quantified the responses of soil bacterial and fungal features to degradation and restoration gradients of the alpine grasslands. Equally, little is known about the relationships between soil microbial communities and soil quality in different succession stages.

The main objective of this study is (i) to reveal how soil quality and microbial indicators respond to the degradation and restoration succession gradients of alpine grassland and (ii) to understand the linkage among these indicators. Following these objectives, we hypothesise that (H1) soil quality will decrease as the degradation of grassland progresses; (H2) soil quality will increase with the increase of the establishment years of artificial grassland; (H3) soil microbial features will have a significant change during the degradation and restoration succession of alpine grasslands. To test these hypotheses, we carried out an *in situ* experiment in the grasslands on the Tibetan Plateau. For degradation gradient, we chose four succession stages, including light degradation, moderate degradation, heavy degradation, and extreme degradation grasslands, respectively. For restoration gradient, we chose artificial grasslands at 1, 5, and 10 years after their establishment, representing the early, middle, and late stages of artificial grassland, respectively.

## Materials and Methods

### Study Sites

The experimental site is located in Maqin County of the Golog Tibetan Autonomous Prefecture of Qinghai Province, China (34°21′–34°31′N, 100°10′–100°57′E, and 3,876 m a.s.l., Supplementary Table 2). The area has an alpine and semi-humid climate. The mean annual temperature is 1.3°C, and summer daily temperature is 8.3°C. Mean annual precipitation ranges from 486.9 to 666.5 mm, occurring mainly from June to September. There is entirely no frost-free period. The dominant genera of vegetation species in the study alpine meadow grassland include *Kobresia* spp., *Polygonum* spp., and *Poa* spp. The soil is sandy loam (40% sand, 40% silt, and 20% clay) according to the soil classification of International Society of Soil Science (Dong et al., 2012). Across most of these areas, the vegetation and soil quality have been degraded by yark and sheep overgrazing (Wang et al., 2015). Extremely degraded areas of alpine grassland are “black-soil lands” (Li et al., 2014).

### Experiment Design

We randomly selected 10 m × 10 m sampling plots in different patches across the degradation and restoration gradients in 2018. Based on the percentage of vegetation cover and plant species we classified the plots into different degradation stages. These stages included light degradation (D1), moderate degradation (D2), heavy degradation (D3), and extreme degradation (D4). Corresponding to >90, 60–90, 30–60, and <30% of healthy grassland, respectively (Supplementary Table 1). D1 was a healthy rangeland dominated by grass and sedges plant species. D2 was exposed to low levels of grazing, and was dominated by grass, sedges, and forbs plant communities. D3 was expose to long-time overgrazing and was mainly dominated by forb plant species while D4 is overgrazed and has turned into “black-soil land” (Supplementary Figure 1; Li et al., 2014). To assess restored grasslands, we used the artificial restored “black-soil land” with *E. nutans* and *P. pratensis* as the main species at 1 year (R1), 5 years (R5), and 10 years (R10) (Supplementary Figure 1). Each sample plot size was 100 m^2^ (10 m × 10 m), and we had four replicates in a completely randomised block design (the geographic coordinates information of the sampling sites is shown in Supplementary Table 2). Each sampling plot contained four subplots (1 m × 1 m), and the distance between the subplots was 50 m, to avoid noisy environmental factors, such as temperature, precipitation, soil type, plant community, and elevation.

### Collection of Soil and Root Samples

Field soil sampling was conducted in July 2018. Soil samples (0–20 cm soil layer) were collected from four quadrats in each plot using bucket auger (5 cm in diameter and 10 cm in depth). Root samples were also taken from the same plots where the soil samples were collected. The samples from each sub-plot were bulked into one composite soil sample. The soil samples were then divided into two parts. One part was air-dried, and sieved using 2 and 0.25 mm mesh sieves to collect soil roots analyse the soil chemical properties. The second part was stored in a refrigerator at 4°C for microbial biomass analysis. From the fresh soil samples, 10 g of soil per sample was stored in a refrigerator at −80°C for DNA extraction and high throughput sequencing. Soil root samples were washed inside a 0.25 mm mesh filter gauze bag and then oven dried at 65°C for 48 h to weigh the root biomass.

### Soil Chemical Analysis and Enzymatic Activity

Soil total nitrogen (TN) was assessed using a semi-micro Kjeldahl digestion procedure (Pruden et al., 1985). Soil total phosphorus (TP) using the ammonium molybdate method; soil total potassium (TK) was investigated by flame photometry; soil organic matter (OM) was determined using WalkleyBlack acid digestion method by K_2_Cr_2_O_7_; Available nitrogen (AN) was determined using the alkaline hydrolysis method (Bao, 2005). The extraction of available phosphorus (AP) was achieved using HCl-NH_4_F, and analysed using the ammonium molybdate method (Wu et al., 2010). Available potassium (AK) was extracted by ammonium acetate (NH_4_OAc) and analysed using the flame atomic absorption spectrometric method (Bao, 2005). Soil pH was determined in a soil: water (1:5) solution using a pH metre and the soil electrical conductivity (EC) was measured using a conductivity metre (Spectrum Technology Inc., United States). Soil bulk density (BD) was measured in the grassland field by the volumetric ring method, and soil moisture (SM) was determined using the oven drying method (Li et al., 2014). We determined six enzymatic activities involved in soil carbon, nitrogen, and phosphorus cycles, which were β-1,4-N-acetylglucosaminidase (NAG), leucine aminopeptidase (LAP), N-acetyl-β-D-glucosaminidase (BG), acid phosphatase (ACP), peroxidase (PER), and polyphenol oxidase (PPO), respectively. NAG, LAP, BG, and ACP were measured by a fluorescent micro-plate enzyme assay (Qi et al., 2016). PPO and PER were measured by using the substrate of l-DOPA in clear micro-plates (DeForest, 2009).

### Analyses of Soil Microbial Biomass and High-Throughput Sequencing

Soil microbial biomass carbon (MBC), microbial biomass nitrogen (MBN), and microbial biomass phosphorus (MBP) were measured by fumigation-extraction method (Vance et al., 1987). We used 16S and ITS ribosomal RNA to study uncultured microbial populations in complex environments (Handelsman, 2009; Oulas et al., 2015).

First, DNA was extracted from 0.5 g of soil using the E.Z.N.A.^®^ Kit (Omega Biotek, Norcross, United States). The V3–V4 regions of the bacteria 16S RNA gene was amplified by PCR using primers 338F/806R (5′-ACTCCTACGGGAGGCAGCAG-3′/5′-GGACTACHVGGGTWTCTAAT-3′) (Yao et al., 2018). While the ITS1 region of fungi was amplified using primers ITS1F/ITS2R (5′-CTTGGTCATTTAGAGGAAGTAA-3′) and (5′-GCTGCGTTCTTCATCGATGC-3′). PCR reactions were performed in triplicate 20-μL mixtures containing 4 μL of 5× FastPfu Buffer, 2 μL of 2.5 mM dNTPs, 0.8 μL of each primer (5 μM), 0.4 μL of FastPfu Polymerase (TransGen Biotech., China), and 10 ng of template DNA. Bacterial and fungal PCR products were pooled separately for sequencing. The PCR amplification of 16S rRNA gene was denatured at 95°C for 3 min, followed by 27 cycles of denaturing at 95°C for 30 s, 30 s annealing at 55°C, extension at 72°C for 45 s, followed by extension at 72°C for 10 min, and ended at 4°C. The PCR amplification conditions of ITS was performed as follows: initial denaturation at 95°C for 3 min, followed by 35 cycles at 94°C for 1 min, 1 min annealing at 51°C, 72°C for 1 min and single extension at 72°C for 10 min. The amplicons were extracted from 2% agarose gels and purified using the AxyPrep DNA Gel Extraction Kit (Axygen Biosciences, United States). Purified amplicons were pooled in equimolar and paired-end sequenced on an Illumina MiSeq PE300 platform/NovaSeq PE250 platform (Illumina, San Diego, CA, United States), according to the standard protocols by Majorbio Bio-Pharm Technology Co. Ltd. (Shanghai, China). Acquired initial sequence used the QIIME (V1.9.1) quality control process to conduct the extraction of high-quality clean tags, the sequences were clustered into operational taxonomic units (OTUs) according to 97% pairwise identity with the USEARCH tool based on the UCHIME algorithm (Edgar et al., 2011).

### Statistical Analysis

Soil nutrients, enzyme activity and microbial parameters were tested by the One-way ANOVA (*P* < 0.05) to compare the significant difference among different degradation or restoration levels using SPSS version 20.0 (IBM Corp., Armonk, NY, United States). ANOVA analysis was separately performed on two groups: one ANOVA for the “degradation group,” and the other ANOVA for the “restoration group.” Sigma Plot 12.5 software (Systat Software Inc., Point Richmond, CA, United States) was used to plot the column graphs. We separately analysed the microbial community structure under degradation and restoration conditions of alpine meadow using R (v.3.3.2 R Core Team, 2019^1^). The Chao1 and Shannon indices (alpha diversity) were analysed using the package “phyloseq.” Bacterial and fungal community beta diversity was quantified by Nonmetric multidimensional scaling (NMDS) analysis based on Bray–Curtis dissimilarities using the “vegan” package. The significance of different factors on bacterial and fungal community was tested using the “adonis” function of the “vegan” package. In addition, bacterial and fungal biomakers across different degradation or restoration gradients were discovered by linear discriminate analysis (LDA) effect size (LefSe^2^) in LDA score >4.0 and *P* < 0.05. Redundancy discriminate analysis (RDA)/Canonical correspondence analysis (CCA) was used to test the relationship between microbial community, enzyme activity and soil quality across degradation/restoration gradients at OTU levels. It is recommended to use RDA when the gradient length is <3 units, CCA when it is >4 units. Nonparametric multivariate of similarities analysis (ANOSIM) was performed using “anosim” function”. One-way ANOSIM (analysis of similarities) was performed to test the effects of degradation and restoration gradients on microbial (bacterial and fungal) communities using “anosim” function. Non-metric multidimensional scaling (NMDS) analysis revealed that the soil samples of different degraded and restored grassland gradients formed distinct clusters in the ordination space. Further, Spearman rank correlation test was employed to determine the correlations between relative abundances of 15 dominant microbial groups and environmental factors across degradation/restoration gradients.

## Results

### Soil Quality and Below-Ground Biomass

Soil nutrients and below-ground biomass varied widely among the different degradation and restoration gradients (Table 1). Compared with D1, the concentration of TN, AN, and OM decreased significantly in D3, TK significant decrease with grassland degradation. Artificial grasslands establishment for 10 years, TN, TP, OM, and AK increased significantly (Table 1). Below-ground biomass (BGB) in D2 (2.8 g m^–2^), D3 (0.7 g m^–2^), and D4 (0.4 g m^–2^) significantly decreased by 57.4, 89.4, and 94.2% compared with that in D1 (6.6 g m^–2^) (*P* < 0.05, Table 1). However, no significant differences in BGB among the three restoration gradients were found. In addition, SM decreased with degradation gradients and increased with restoration gradients, but the changes in BD were opposite to that in SM. SM and BD between D1 and D4 and between R1 and R10 were significantly different.

**TABLE 1:** Descriptive statistics for soil chemistry properties and below-ground biomass in alpine grassland in light degradation (D1), moderate degradation (D2), heavy degradation (D3), and extreme degradation (D4) stages, and after restoration for 1 (R1), 5 (R5), and 10 (R10) years.

	D1	D2	D3	D4	R1	R5	R10
TN (g kg^–1^)	5.29 ± 0.78a	4.41 ± 0.67ab	3.31 ± 0.11b	4.47 ± 0.35ab	2.41 ± 0.18b	2.56 ± 0.11b	3.60 ± 0.41a
TP (g kg^–1^)	0.62 ± 0.06b	0.56 ± 0.02b	0.62 ± 0.05b	0.76 ± 0.01a	0.55 ± 0.04b	0.57 ± 0.01ab	0.65 ± 0.02a
TK (g kg^–1^)	18.63 ± 1.43a	15.64 ± 0.15b	15.61 ± 0.08b	14.99 ± 0.47b	15.60 ± 0.52b	16.72 ± 0.10a	16.18 ± 0.20ab
OM (g kg^–1^)	100.70 ± 9.36a	85.47 ± 14.02a	55.30 ± 3.44b	79.81 ± 4.02ab	44.21 ± 3.19b	45.55 ± 2.15b	63.54 ± 8.20a
AN (mg kg^–1^)	188.65 ± 21.40a	147.70 ± 18.72ab	91.00 ± 26.14b	168.47 ± 20.86a	89.60 ± 8.22b	124.25 ± 9.42a	123.20 ± 13.75ab
AP (mg kg^–1^)	6.14 ± 0.28a	6.53 ± 0.37a	7.21 ± 0.64a	7.58 ± 0.58a	13.57 ± 9.50a	9.49 ± 3.43a	6.58 ± 0.89a
AK (mg kg^–1^)	175.00 ± 1.59a	160.70 ± 4.12a	153.23 ± 24.56a	139.37 ± 33.08a	89.35 ± 13.14b	129.18 ± 25.51ab	167.23 ± 18.85a
pH	6.65 ± 0.06a	7.14 ± 0.22a	7.17 ± 0.17a	7.08 ± 0.25a	8.13 ± 0.06a	7.79 ± 0.28ab	7.28 ± 0.31b
EC (μS cm^–1^)	49.78 ± 7.47b	98.03 ± 22.55a	95.83 ± 10.04a	134.17 ± 12.75a	90.80 ± 6.36a	51.43 ± 8.10b	100.73 ± 4.69a
SM (%)	30.45 ± 2.63a	26.29 ± 1.36ab	28.33 ± 3.50ab	20.97 ± 1.43b	17.69 ± 0.52b	19.83 ± 0.84b	24.01 ± 0.89a
BD (g cm^–3^)	0.93 ± 0.04b	1.13 ± 0.06ab	1.29 ± 0.06a	1.24 ± 0.13a	1.47 ± 0.03a	1.53 ± 0.03a	1.34 ± 0.05b
BGB (g m^−2^)	6.63 ± 1.23a	2.83 ± 1.43b	0.70 ± 0.60b	0.38 ± 0.19b	0.12 ± 0.06a	0.31 ± 0.06a	0.28 ± 0.07a

*Means ± standard errors. Data in a row without shared letters (“degradation group” and “restoration group” was separately performed) indicates significant differences at p < 0.05. TN, total nitrogen; TP, total phosphorus; TK, total potassium; OM, organic matter; AN, available nitrogen; AP, available phosphorus; AK, available potassium; EC, electrical conductivity; SM, soil moisture; BD, bulk density; BGB, below-ground biomass.*

### Soil Microbial Biomass and Enzymes

**FIGURE 1:**
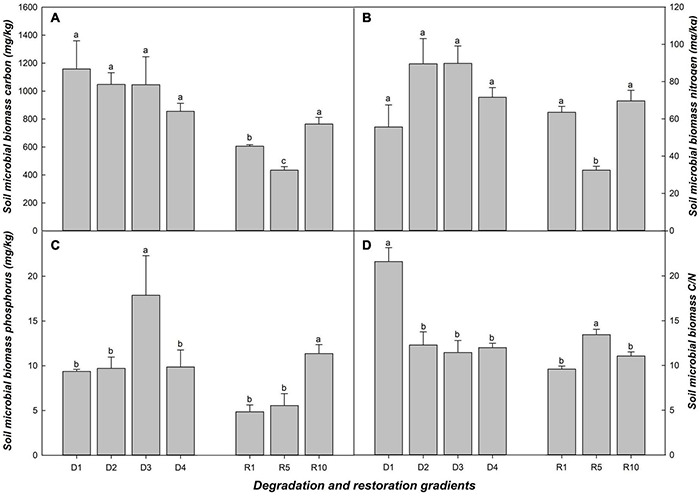
Soil microbial biomass in alpine grasslands at light degradation (D1), moderate degradation (D2), heavy degradation (D3), and extreme degradation (D4) stages, and after restoration for 1 (R1), 5 (R5), and 10 (R10) years. Panels **(A–D)** shows soil microbial biomass carbon, nitrogen, phosphorus, and soil microbial biomass carbon: nitrogen in different grasslands, respectively. Lowercase letters represent the difference among degradation/restoration gradients (*n* = 4 for the degradation gradient and *n* = 3 for the restoration gradient, *p* < 0.05).

Soil microbial biomass carbon (MBC) and soil microbial biomass nitrogen (MBN) were more sensitive to restoration than to degradation process (Figures 1A,B). In grassland degradation, the changes in MBC and MBN were not obvious, but MBC/MBN ratio in D1 was significantly higher than that in D2, D3 and D4 (Figures 1A,B,D). Regarding grassland restoration, MBC, MBN and soil microbial biomass phosphorus (MBP) reached max in R10; MBC and MBN in R1 and R10 were significantly higher than those in R5, but MBC/MBN in R5 was significantly higher than in R1 and R10 (Figure 1).

**FIGURE 2:**
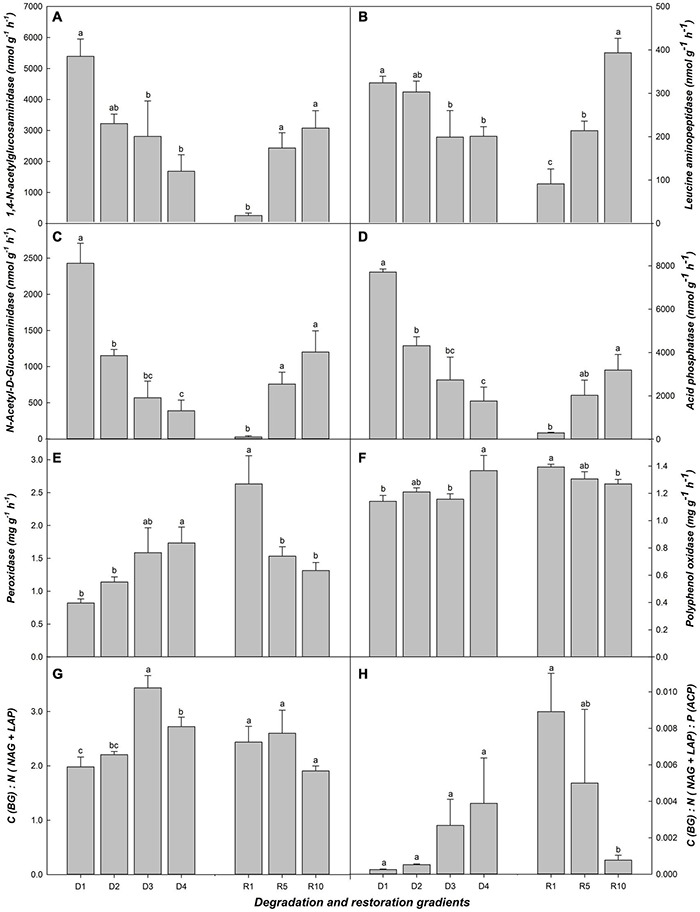
Soil enzymes related to microbial biomass in alpine grassland at light degradation (D1), moderate degradation (D2), heavy degradation (D3), and extreme degradation (D4) stages, and after restoration for 1 (R1), 5 (R5), and 10 (R10) years. Panels **(A–F)** shows BG, 1,4-N-acetylglucosaminidase; LAP, Leucine aminopeptidase; NAG, N-acetyl-D-glucosaminidase; ACP, Acid phosphatase; PER, Peroxidase; PPO, Polyphenol oxidase in different grasslands, respectively. BG related to carbon cycle, NAG and LAP related to nitrogen cycle, ACP related to phosphorus. Panels **(G)** shows the ration of BG to NAG + LAP. Panels **(H)** shows rations of BG: NAG + LAP: ACP. Lowercase letters represent the difference among degradation/restoration gradients (*n* = 4 for the degradation gradient and *n* = 3 for the restoration gradient, *p* < 0.05).

Overall, BG related to carbon cycle, LAP, and NAG related to nitrogen cycle, ACP related to phosphorus cycle, increased significantly after 10 years of grassland restoration, and decreased significantly from D1 to D3 (Figures 2A–D). The responses of PER and PPO were opposite to those of the above enzymes (Figures 2E,F). The transition stages of succession (e.g., D2, D3, and R5) were often less responsive to soil enzyme activity than the initial and final stages (e.g., D1 and D4, R1 and R10) (Figure 2). C (BG): N (NAG+LAP) ratio, and C (BG): N (NAG+LAP): P (LAP) ratio not well represent the soil microbial biomass of grassland succession (Figures 2G,H).

### Bacterial and Fungal Community Compositions

**FIGURE 3:**
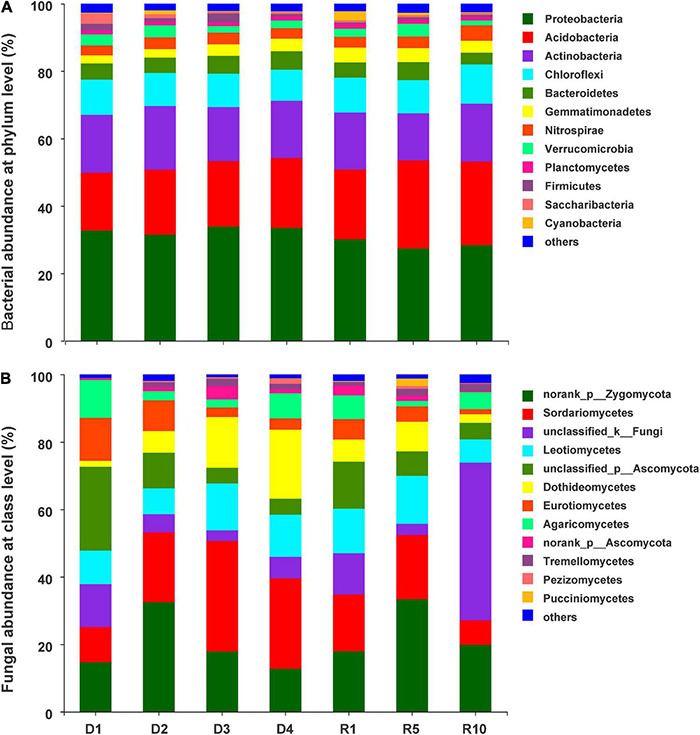
The distribution of abundant taxa with relative abundances > 1% in at least one sample. **(A)** Soil bacterial community at phylum level; **(B)** soil fungal community at class level. D1, D2, D3, and D4 represent light, moderate, heavy, and extreme degradation stages; R1, R5, and R10 represent artificial restoration for 1, 5, and 10 years, respectively.

In total, 1,611,248 bacterial sequences and 1,978,412 fungal sequences were obtained from the 28 soil samples. This generated 4,310 bacterial operational taxonomic units (OTUs) and 2,788 fungal OTUs at 97% similarity. Most of bacterial sequences belonged to the phyla Proteobacteria (31.1%), Acidobacteria (21.2%), Actinobacteria (16.7%), and Chloroflexi (10.16%), accounting for 79.1% of all sequences (Figure 3A). In addition, Bacteroidetes (4.8%), Gemmatimonadetes (3.5%), Nitrospirae (3.4%), Verrucomicrobia (2.7%), Planctomycetes (1.2%), Firmicutes (1.2%), and Saccharibacteria (1%) presented relative abundances >1%. The fungal phylum was dominated by Ascomycota (58.1%), Zygomycota (21.3%), and Basidiomycota (7.5%), respectively. At the class level, the dominant fungi with abundances over 5% included Sordariomycetes (19.1%), Leotiomycetes (11.2%), Dothideomycetes (8.8%), Eurotiomycetes (5.8%), and Agaricomycetes (5.3%) (Figure 3B).

**FIGURE 4:**
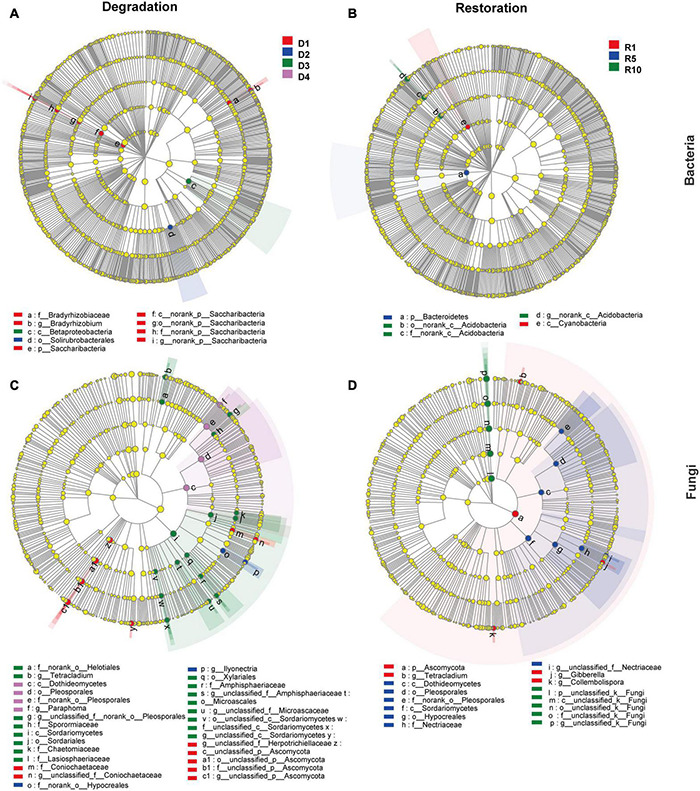
Cladograms generated by the LEfSe analysis revealing differences in the clade at the LDA threshold of 4.0 [**(A,B)** bacterial community; **(C,D)** fungal community]. D1, D2, D3, and D4 represent alpine grassland in light, moderate, heavy, and extreme degradation stages, respectively; R1, R5, and R10 represent alpine grassland after restoration for 1, 5, and 10 years, respectively. Small circles and shading with different colors in the diagram illustrate the taxa that are enriched in the different degradation and restoration stages.

The LEfSe analysis revealed that nine bacterial clades (affiliating with phyla Actinobacteria, Proteobacteria, and Saccharibacteria) and 29 fungal clades (affiliating with phyla Ascomycota) were susceptible to four degraded succession stages (Figures 4A,C). At bacterial phyla level, the relative abundance of Saccharibacteria changed significantly along degraded successional stages (*P* < 0.05), i.e., the highest abundance occurred in D1 (3%), followed by D2 (1.1%), and the lowest was in D3 (0.5%) and D4 (0.6%). However, Sordariomycetes and Dothideomycetes followed the opposite distribution pattern at the fungal class level (Figure 4C). The relative abundances of Dothideomycetes followed the order of D4 (20.4%) > D3 (15.1%) > D2 (6.4%) > D1 (1.8%) (Figure 4C).

Consistently, five bacterial clades (affiliating with phyla Actinobacteria, Bacteroidetes, and Cyanobacteria) and 16 fungal clades (affiliating with the phylum Ascomycota) shown significant variances among the three restoration gradients at the LDA threshold of 4.0 (Figures 4B,D). At the bacterial phlum level, Bacteroidetes (bacteria) and Ascomycota (fungi) were less abundant in R10 than in R1 and R5.

### Bacterial and Fungal Community Diversity

The alpha-diversity of bacterial and fungal communities showed opposite trends between grassland degradation and restoration successional stages (Table 2). The degradation significantly increased microbial shannon and Chao1 indices, e.g., the Chao1 index of fungi followed the order of D4 (697.1) > D3 (660.5) > D2 (643.3) > D1 (443.7) (Table 2). Conversely, the fungal Shannon and Chao1 indices of revegetated grassland significantly decreased from R5 to R10 (*P* < 0.05). The bacterial Chao1 index also indicated similar trends, whereas the bacterial shannon index did not differ significantly among restoration successional stages.

**TABLE 2:** Diversity indices of soil bacterial and fungal communities in alpine grasslands in light degradation (D1), moderate degradation (D2), heavy degradation (D3), and extreme degradation (D4) stages, and after restoration for 1 (R1), 5 (R5), and 10 (R10) years.

Treatment	Bacteria		Fungi	
	Shannon	Chao1	Shannon	Chao1
D1	6.40 ± 0.07c	2521.10 ± 54.46b	3.05 ± 0.16b	443.74 ± 33.81b
D2	6.60 ± 0.04b	2945.78 ± 80.92a	3.36 ± 0.54ab	643.34 ± 106.37a
D3	6.71 ± 0.04ab	2924.23 ± 68.72a	4.25 ± 0.08a	660.49 ± 31.68a
D4	6.77 ± 0.01a	3107.03 ± 69.38a	3.92 ± 0.29ab	697.05 ± 39.04a
R1	6.60 ± 0.06a	2945.45 ± 57.87b	4.09 ± 0.03a	612.96 ± 32.23ab
R5	6.73 ± 0.05a	3130.35 ± 43.99a	4.04 ± 0.10a	685.43 ± 32.45a
R10	6.64 ± 0.03a	2906.09 ± 36.91b	2.79 ± 0.65b	502.24 ± 44.04b

*Data in a row without shared letters (“degradation group” and “restoration group” was separately performed) indicates significant differences at p < 0.05.*

**FIGURE 5:**
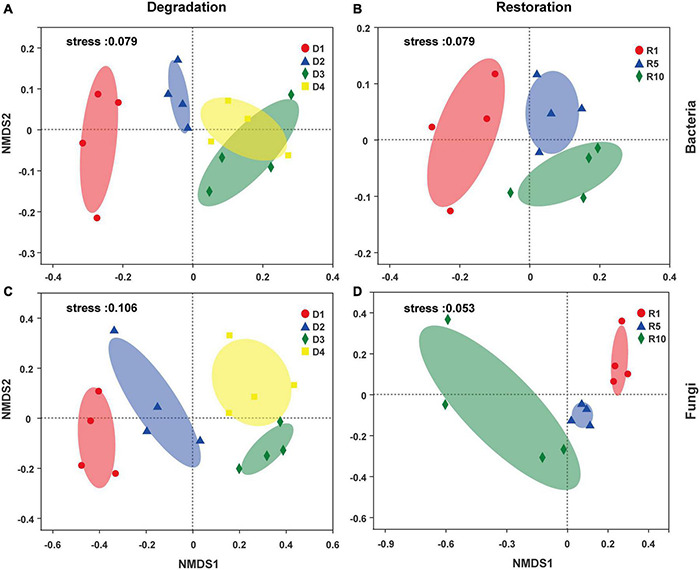
Nonmetric multidimensional scaling (NMDS) plots of microbial community across degradation/restoration gradients on OTU levels [**(A,B)** bacterial community; **(C,D)** fungal community]. D1, D2, D3, and D4 represent alpine grassland in light, moderate, heavy, and extreme degradation stages, respectively; R1, R5, and R10 represent alpine grassland after restoration for 1, 5, and 10 years, respectively.

Non-metric multidimensional scaling (NMDS, based on Bray–Curtis distance) plots observed that both bacterial and fungal community structure were dramatically different among grassland degradation or restoration successional stages (Figure 5). ANOSIM further confirmed that the degradation successional stage was the main factor shaping bacterial and fungal community structures (Figures 5A,C, R = 0.62, *P* < 0.001; R = 0.66, *P* < 0.001, respectively). Similarly, the discernible differences in bacterial and fungal communities subjected to the three restoration successional stages were also present (Figures 5B,D, ANOSIM, R = 0.58, *P* < 0.001; R = 0.53, *P* < 0.001, respectively).

Based on the network analysis of significant correlation (Spearman’s correlation coefficients, R^2^> 0.5, *P* < 0.05), the co-occurrence patterns of microorganisms (fungi and bacteria) in different degraded and artificially restored grassland gradients were determined (Supplementary Figure 2). The number of bacteria-bacteria links in heavy and extreme degraded grasslands (D3+D4, 125) was significantly lower than that in light and moderate degraded grasslands (D1+D2, 159, Supplementary Figures 2A,B). The network analysis of fungi also showed a similar trend, i.e., the connection number in light and moderate degraded grasslands was nearly 2 times that in heavy and extreme degraded grasslands (135 vs. 76, Supplementary Figures 2D,E). In addition, the numbers of bacteria-bacteria and fungi-fungi links were increased in artificial restoration grasslands (R5+R10, 168, 244, Supplementary Figures 2C,F).

### Relationship Between Microbial Community and Soil Features

**FIGURE 6:**
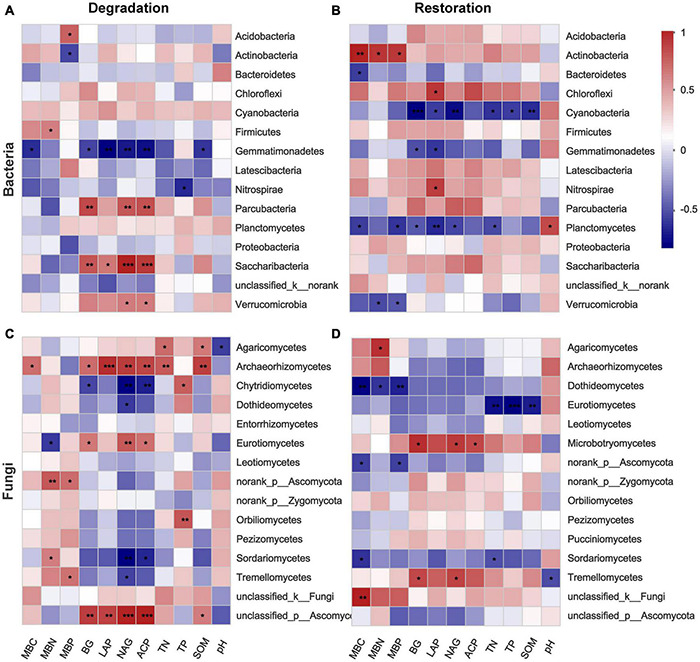
Heatmap of Spearman’s rank correlation coefficients between relative abundance of 15 dominant microbial groups and environmental factors across degradation/restoration gradients based on Bray–Curtis distances [**(A,B)** bacterial community; **(C,D)** fungal community]. BG, 1,4-N-acetylglucosaminidase; LAP, leucine aminopeptidase; NAG, N-acetyl-D-glucosaminidase; ACP, acid phosphatase. ***p ≤ 0.001, **0.001 < *p* ≤ 0.01, *0.01 < *p* ≤ 0.05.

Spearman correlation heatmap showed that soil microbial biomass and enzymes significantly influenced bacteria and fungi abundance (Figure 6). Overall, soil enzyme factors were associated with the abundance of bacteria and fungi in the four degradation successional stages. LAP, BG, NAG, and ACP showed significant negative correlations with Gemmatimonadetes, but was positively correlated with Verrucomicrobia, Saccharibacteria and Parcubacteria at the phylum level (Figure 6A). NAG showed a negative correlation with 4 classes, and a positive correlation with three classes, which was the highest effect of an enzyme on fungi (Figure 6C). Regarding the three restoration successional stages, Actinobacteria was positively related to MBC, MBN, and MBP (*P* < 0.05), whereas Cyanobacteria was negatively related to TN, TP, and OM (*P* < 0.05) at the bacterial phylum level (Figure 6B). Furthermore, Dothideomycetes was negatively related to MBC, MBN, and MBP (*P* < 0.05), and Eurotiomycetes was negatively related to TN, TP, and OM (*P* < 0.01) at the fungal class level (Figure 6D).

**FIGURE 7:**
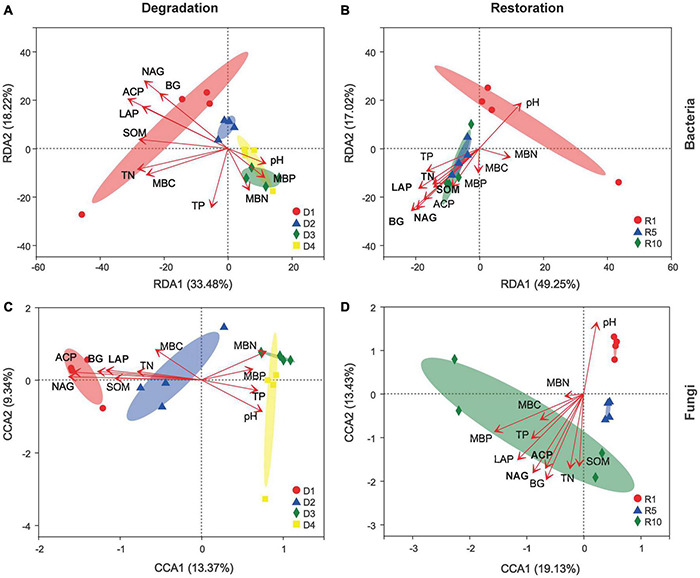
Redundancy discriminate analysis (RDA)/Canonical correspondence analysis (CCA) ordination diagram of microbial community across degradation/restoration gradients on OTU levels **(A)** RDA of soil bacterial community composition across degradation gradients; **(B)** RDA of soil bacterial community composition across restoration gradients; **(C)** CCA of soil fungal community composition across degradation gradients; **(D)** CCA of soil fungal community composition across restoration gradients. Gradient length along the first ordination axis is used for selecting RDA or CCA; It is recommended to use RDA when the gradient length is <3 units, CCA when it is >4 units. D1, D2, D3, and D4 represent alpine grassland in light, moderate, heavy, and extreme degradation stages, respectively; R1, R5, and R10 represent alpine grassland after restoration for 1, 5, and 10 years, respectively. BG, 1,4-N-acetylglucosaminidase; LAP, leucine aminopeptidase; NAG, N-acetyl-D-glucosaminidase; ACP, acid phosphatase.

Possible linkages among microbial community structures, soil nutrients and enzyme activities were further uncovered based on RDA/CCA analysis (Figure 7). In degradation successional stages, the variations in bacterial community structure (the joint probability of the first two axes was 51.7%) could be explained more than that in fungal community structure (22.71%), and all sites were separated by the two ordination axes in view of degradation stages (Figures 7A,C). Soil enzyme activities, including BG (R^2^ = 0.53, *P* = 0.002), LAP (R^2^ = 0.56, *P* = 0.006), NAG (R^2^ = 0.81, *P* = 0.001), and ACP (R^2^ = 0.78, *P* = 0.001), were significantly correlated with bacterial community structure. Meanwhile, the RDA/CCA analysis explains 66.3% of the variance in bacterial community and 32.6% in fungal community in the four degradation successional stages (Figures 7B,D). BG produced the strongest linkage between bacterial and fungal community structure.

## Discussion

### Responses of Soil Quality to Degradation and Restoration

Soil quality is disturbed by external factors that result in plants composition and soil environment changes during the different successional stages of alpine grasslands (Dong et al., 2012; Li et al., 2014). Our results validate our first hypothesis that soil quality decreases with the degradation of grassland and increases with the increase of the establishment years of artificial grasslands. We also found disparities in the soil quality responses to degradation and restoration gradients. The concentration of TN, OM showed a decreasing trend along the degradation gradient. These results are consistent with those of a meta-analysis over a global scale, which showed that continuous grazing significantly reduced soil organic carbon (SOC, SOC = OM/1.724) and TN compared with a no-grazing regime (Byrnes et al., 2018; Wang et al., 2020). The changes in phosphorus content, however, was not obvious. Most studies have reported that phosphorus content decreases significantly during degradation and considered to be a result of grassland overutilisation (Zhou et al., 2005, 2016; Wu et al., 2014). These different observations could mainly result from the fact that phosphorus is a sedimentary element with weak mobility and large spatial differences (Halbfaß and Grunewald, 2003; Wang et al., 2020). The concentrations of TN, AN, TK, and OM had minimum values in D3 and were significantly lower than those in D1 (Table 1), indicating that heavy degraded grassland is the critical point of the change of soil nutrient content. Grazing intensity negatively affected carbon and nitrogen storage (Li et al., 2014), but soil nutrient contents increased with the restoration gradient for TN, TP, and OM in R10 were significantly higher than those in R1 (Table 1), thereby validating our second hypothesis. These contradicts earlier results of an artificially restored grassland in the Tibetan Plateau by Li et al. (2014), who reported that soil nutrients substantially decreased with increasing restoration after 5, 7 and 9 years, respectively. However, our results corroborate those of Wu et al. (2010), who reported that 6 years *E. nutans* artificial grassland significantly increased soil carbon. The main reason underlying these observations could be the differences in the number of years of these artificially restored grasslands and the types of forage seed used. It is well known that long-term overgrazing leads to soil compaction, reduced soil porosity, soil infiltration, resulting in an increase in soil bulk density and decrease in soil moisture (Holt, 1997). Our results also showed that BD increased with the degradation gradient and decreased with the restoration gradient. However, the SM changes were opposite to those of BD. Soil moisture can be altered by changes in vegetation (Dong et al., 2012), with the increase of artificial grassland establishment years, SM showed a significant level between R1 and R10.

As one of the crucial indicators of soil quality, soil enzymatic activity plays an important role in soil alpine grassland ecological processes and is closely related to the proliferation of soil microbial communities (Gómez-Sagasti et al., 2012; Li et al., 2019). Four soil enzymes related to MBC, MBN, and MBP all showed significant consistency each other. BG is related to MBC, LAP, and NAG are related to MBN, and ACP is related to MBC (Xu et al., 2018). The changes in soil enzymes in our study also validated our hypothesis. Overall, the values of NAG, LAP, BG, and ACP decreased along the degradation gradient and increased along the restoration gradient (Figure 2). The C (BG): N (NAG+LAP): P (ACP) ratio increased with degradation, as we observed that the ratios in the light and moderate degradation stages were lower than those in the heavy and extreme degradation stages. However, the ratio decreased across the restoration gradient. We observed no significant difference in the C (BG): N (NAG+LAP): P (ACP) ratio between R5 and R1 or R10, but the ratio of R10 was significantly higher than that of R1. In this study, the division of grassland gradients was based on the vegetation characteristics, especially vegetation coverage (Supplementary Figure 1 and Table 1). Whether grassland succession stages can be classified based on the grassland ecological significance according to the C (BG): N (NAG+LAP): P (ACP) ratio is worth being considered in future. MBC, MBN and MBP did not show the same regularity in the different degradation and restoration stages, but soil enzymes related to them showed more remarkable and coincident changes. This indicates that the sensitivity of enzymatic activity to alpine grassland succession is higher than that of microbial biomass (Si et al., 2015; Pu et al., 2016). Furthermore, which could provide a new research direction for the study of microbial changes in alpine grassland ecosystems.

### Changes of Soil Microbial Composition Across Degradation and Restoration Gradients

Bacterial communities were overwhelmingly dominated by Proteobacteria, Acidobacteria and Actinobacteria in the different grassland successional stages (Figure 3A). Ascomycota was the dominant fungal phyla were, accounting for an average of 63.5% of the total fungal sequences. These findings were consistent with Ambardar et al. (2016). This observation could be explained by the Ascomycota’s physiological ability to break down the biochemical structure of plant litter in the degraded alpine steppes. Ascomycota was the predominant fungal phylum, playing an important role in the litter decomposition process and nutrient cycles (Hartmann et al., 2017). In addition, Proteobacteria plays an important role in energy metabolism e.g., breaking down inorganic and organic compounds and capturing energy from light (Mukhopadhya et al., 2012). Meanwhile, Actinobacteria and Acidobacteria represent a variety of saprophytic organisms that play an important role in the decomposition of recalcitrant carbon, especially with strong metabolic capacity at low temperatures (Yergeau et al., 2010). Goordial et al. (2017) conveyed that Actinobacteria was the main phylum in permafrost bacterial communities from the McMurdo Dry Valleys of Antarctica. Above all, these results confirmed that Proteobacteria, Acidobacteria, Actinobacteria, and Ascomycota have a good adaptability to soil properties and vegetation changes in degraded alpine grasslands on the Tibetan Plateau.

Furthermore, the LEfSe analysis revealed that there were 21 biomarkers (including bacteria and fungi) in the degradation stages and 38 biomarkers in the restoration successional stages. These findings suggest that microbes that mediate special ecological functions, such as matter degradation and carbon mineralisation are better survivors in the degraded alpine grassland (Bier et al., 2015; Che et al., 2015). For instance, at the bacterial phylum level, the relative abundance of Bacteroidetes differed significantly among restoration successional stages (*P* < 0.05). Similar results were also reported by Gao et al. (2021). Bacteroidete are considered to participate in the degradation of nitrite oxidation and carbon metabolism (Fang et al., 2018). Notably, the most abundant fungal biomarkers belonged to Ascomycota. The relative abundance of Ascomycota showed an opposite trend between grassland degradation and restoration successional stages, i.e., the abundance increased with degradation stages, but diminished with restoration years (Figures 4C,D). The average abundance of Ascomycota reached its highest level in the heavily degraded stage when the grass felt layer had formed (Figure 3). Ascomycota forms ascospores in their sexual stage. Most ascomycetes are terrestrial, and their nutritional methods included saprophytic, parasitic, and symbiotic nutrition. Saprophytic ascomycetes can cause decomposition of plant residue and breakdown lignin (Jaklitsch et al., 2014). Therefore, the average Ascomycota abundance increased in the heavy and extreme degradation stages, suggesting that saprophytic ascomycetes promote the decomposition of dead roots and animal residues in the grass felt layer.

### Dynamics of Microbial Community Diversity Across Degradation and Restoration Gradients

Understanding bacterial and fungal communities’ response to degradation and restoration gradients is critical to nutrients cycling and ecosystem functioning. In this study, shifted microbial community composition and structure were found in the different degradation and restoration gradients. Overall, the alpha diversity of bacteria and fungi increased with the degradation gradient, but decreased with the restoration gradient. Similar findings were reported by Gao et al. (2021) that bacterial alpha diversity in 1–4 years revegetated grasslands was much higher than that in 10–18 years revegetated grasslands. Hu et al. (2019) also showed that the diversity of bacteria in early revegetated successional stages (4-year) was higher than that of late successional stage (8- and 12-year). For successional stages, Che et al. (2019) reported that degraded patch formation significantly increased fungal alpha diversity by approximately 40%. The observed significant response of soil bacterial and fungal alpha diversity to degradation and restoration gradients could be explained by two aspects. Firstly, soil pH is an important environmental factor that shapes microbial communities (Fierer, 2017) and it frequently changes under different degradation and restoration gradients (Table 1; Lennon and Houlton, 2017). Many studies have reported a negative correlation between soil pH and microbial alpha diversity under different degradation and restoration habitats, which is consistent with our results (Figure 7; Tripathi et al., 2018; Guo et al., 2019). Secondly, reduced vegetation cover in heavily degraded grasslands, promotes erosion and topsoil roughness and increases light availability, and hence advances the competitiveness of aerobiotic microbes and photoautotrophic microbes (Lin et al., 2015; Zhang et al., 2017).

According to NMDS and RDA results, the bacterial and fungal community structures were distinct in different degradation and restoration gradients (Figure 5). This result corroborates earlier studies that reported significant microbial communities’ differences in the degradation or restoration succession process of alpine steppe in the Tibetan Plateau (Li et al., 2016; Guo et al., 2019; Zhou et al., 2019). Compared with light and moderate degradation (D1+D2), heavy and extreme degradation (D3+D4) significantly reduced the interaction between microorganisms, while artificial restoration significantly increased their interaction (Supplementary Figure 2). These differences could result from the differences in vegetation characteristics and soil properties among the degraded and restored grassland. Degraded grassland restricts plant growth and leads to physical damage to soil structure and nutrient status, thus further accelerating soil degradation (Zhou et al., 2019). Zhang et al. (2014) suggested that alpine steppe degradation would significantly alter bacterial community structure due to the reduced availability of microbial matrix induced by the reduced input of litter. In the restored grassland (R5+R10), microbial interactions were enhanced, resulting in a more complex network of interactions. The reason may be that the soil organic matter content is higher and the composition is more diverse in the restored grassland, thus improving soil quality (Gao et al., 2021).

## Data Availability Statement

Publicly available datasets were analysed in this study. This data can be found here: https://login.majorbio.com/login, twwangdj@163.com, m6XbU2.

## Author Contributions

DW performed the experiments, analysed the data, contributed to reagents, materials, and analysis tools, prepared figures and/or tables, authored or reviewed the drafts of the manuscript, and approved the final draft. LM, YS, and ZZ performed the experiments, analysed the data, authored or reviewed drafts of the manuscript, approved the final draft. PC and BY contributed to reagents, materials, and analysis tools, authored or reviewed drafts of the manuscript, and approved the final draft. SD, JW, GS, and XM contributed to reagents, materials, and analysis tools and approved the final draft. DN and FL improved the qualities of the manuscript and approved the final draft. HZ conceived and designed the experiments, analysed the data, contributed to reagents, materials, and analysis tools, prepared figures and/or tables, reviewed drafts of the manuscript, and approved the final draft. ZM and JZ provided ideas for data analysis, plotting, and contributed to supervision. All authors contributed to the article and approved the submitted version.

## Conflict of Interest

The authors declare that the research was conducted in the absence of any commercial or financial relationships that could be construed as a potential conflict of interest.

## Publisher’s Note

All claims expressed in this article are solely those of the authors and do not necessarily represent those of their affiliated organizations, or those of the publisher, the editors and the reviewers. Any product that may be evaluated in this article, or claim that may be made by its manufacturer, is not guaranteed or endorsed by the publisher.

## Footnotes

1
https://www.r-project.org/

2
http://huttenhower.sph.harvard.edu/galaxy/

## Abbreviations

TN: soil total nitrogen (g kg^–1^)
TP: soil total phosphorus (g kg^–1^)
TK: soil total potassium (g kg^–1^)
OM: soil organic matter (g kg^–1^)
AN: soil available nitrogen (mg kg^–1^)
AP: soil available phosphorus (mg kg^–1^)
AK: soil available potassium (mg kg^–1^)
EC: soil electric conductance (uS cm^–1^)
SM: soil moisture (%)
BD: soil bulk density (g cm^–3^)
BGB: below-ground biomass (g m ^−2^)
MBC: soil microbial biomass carbon (mg kg^–1^)
MBN: soil microbial biomass nitrogen (mg kg^–1^)
MBP: soil microbial biomass phosphorus (mg kg^–1^)
BG: β -1,4-N-acetylglucosaminidase (nmol g^–1^ h^–1^)
LAP: leucine aminopeptidase (nmol g^–1^ h^–1^)
NAG: N-acetyl -D-glucosaminidase (nmol g^–1^ h^–1^)
ACP: acid phosphatase (nmol g^–1^ h^–1^)
PER: peroxidase (mg g^–1^ h^–1^)
PPO: polyphenol oxidase (mg g^–1^ h^–1^)
OTUs: operational taxonomic units
D1: light degraded grassland
D2: moderate degraded grassland
D3: heavy degraded grassland
D4: extreme degraded grassland
R1: grassland after restoration 1 year
R5: grassland after restoration 5 years
R10: grassland after restoration 10 years.
