# Supplementary material for: Responses of Soil Microbial Metabolic Activity and Community Structure to Different Degraded and Restored Grassland Gradients of the Tibetan Plateau
Source: Front Plant Sci. 2022 Apr 8;13:770315. doi: 10.3389/fpls.2022.770315 (PMC9024238; doi:10.3389/fpls.2022.770315)
Supplement: Supplementary file 1 [file Data_Sheet_1.docx]

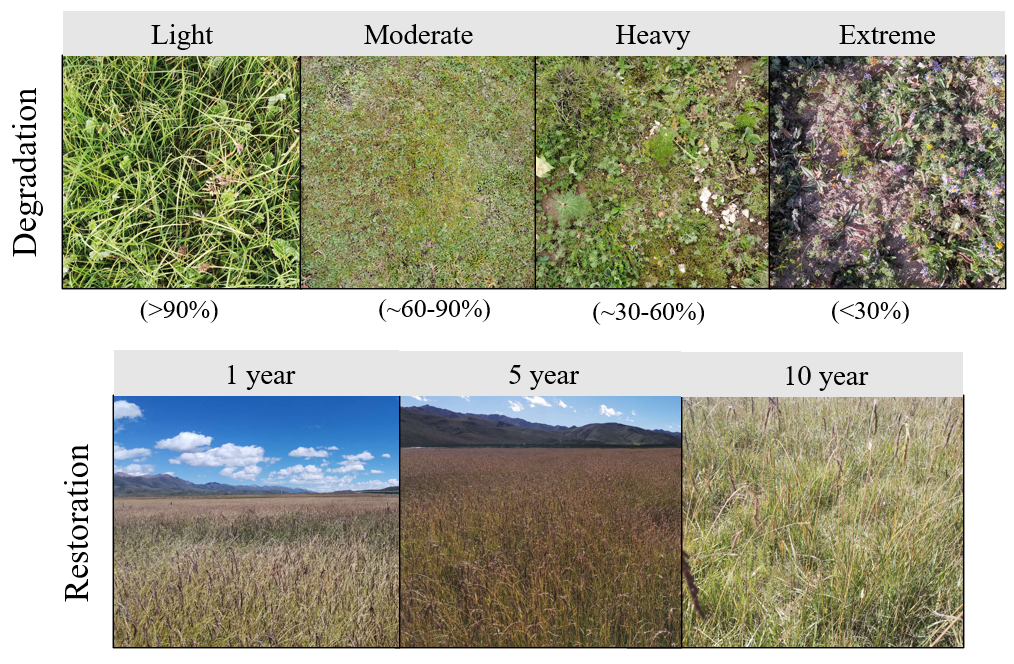
**Fig S1** Photos of grassland in different degradation and restoration stages.


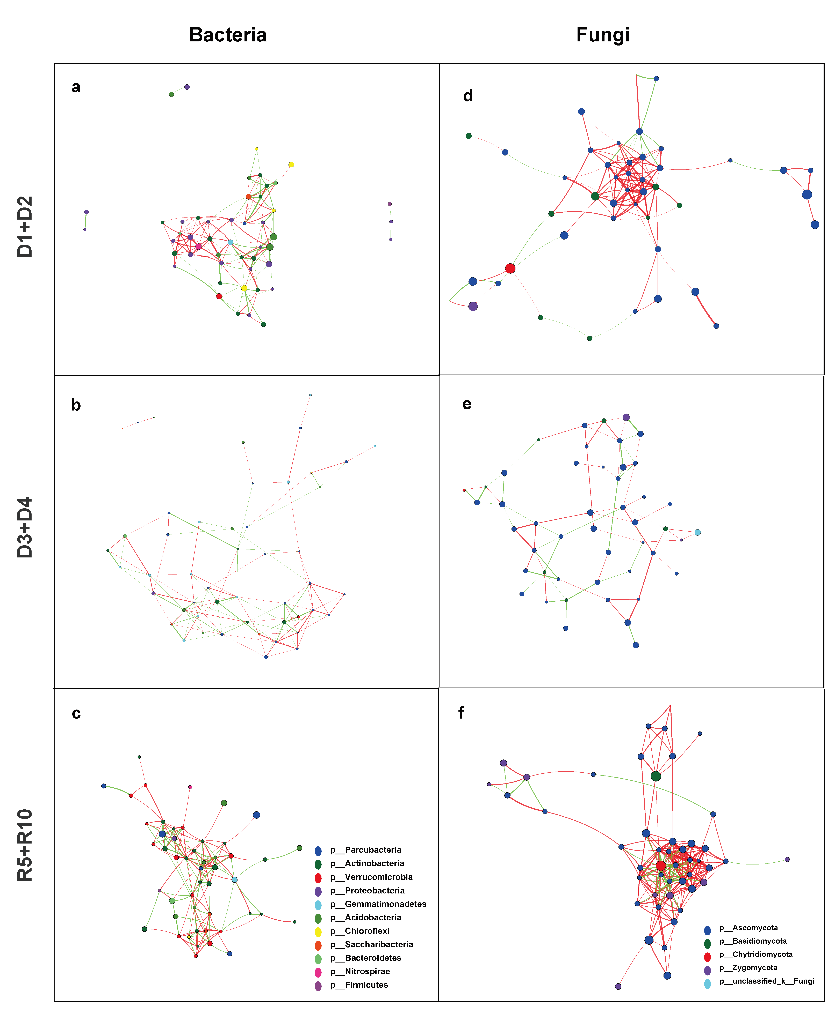


**Fig S2** The network among soil microbial taxa in different degradation and restoration gradients based on the network analysis of significant correlation (Spearman's correlation coefficients, R^2^ > 0.5, p < 0.05) (D1+D2: the light and moderate degraded grasslands, D3+D4: heavy and extreme degraded grasslands, R5+R10: the artificially restored grasslands in 5 and 10 years). The size of each node is proportional to betweenness centrality scores. The line between each pair of nodes represents strong positive (red) or negative (blue) interaction. The thickness of the line indicates the strength of the correlation.

**Table S1** Classification of grassland degradation (Liu et al., 2018, Wang et al., 2021).

| Degradation gradients | Vegetation cover (%) | Pasture quality |
| --- | --- | --- |
| Light degraded grassland (L) | > 90 | Good |
| Moderate degraded grassland (M) | 60~90 | Bad |
| Heavy degraded grassland (H) | 30~60 | Very bad |
| Very heavy degraded grassland (V) | < 30 | Extremely bad |

**Table S2** Location and altitude of the degraded and restored alpine grasslands in Maqin County, Qinghai, China, which are included in this study.

| Treatment | Latitude | Longitude | Altitude (m) |
| --- | --- | --- | --- |
| Light degraded grassland | 34°21'18"N | 100°28'54"E | 3940 |
| Moderate degraded grassland | 34°31'44"N | 100°57'77"E | 4106 |
| Heavy degraded grassland | 34°21'17"N | 100°28'47"E | 3950 |
| Extreme degraded grassland | 34°26'24"N | 100°19'12"E | 3797 |
| Grassland in restoration after 1 year | 34°23'24"N | 100°17'24"E | 3791 |
| Grassland in restoration after 5 years | 34°28'12"N | 100°10'47"E | 3787 |
| Grassland in restoration after 10 years | 34°25'48"N | 100°15'36"E | 3759 |
